# Anticancer Activity of Schiff Base Metal Complexes Against MCF-7 Breast Cancer Cell Line

**DOI:** 10.3390/ijms27020678

**Published:** 2026-01-09

**Authors:** Justyna Samaszko-Fiertek, Barbara Dmochowska, Janusz Madaj

**Affiliations:** Faculty of Chemistry, University of Gdansk, Wita Stwosza 63 Street, 80-308 Gdansk, Poland; basia.dmochowska@ug.edu.pl

**Keywords:** Schiff bases, metal complex, anticancer activity, MCF-7, breast cancer

## Abstract

According to the World Health Organization, breast cancer is the cancer that affects the largest number of people each year, especially women. Millions of women are diagnosed with it each year, and hundreds of thousands die from it. Research into new types of drugs, including metal complexes, including those containing tetradentate Schiff bases as ligands, offers a chance to reduce this number. Various cell lines are being used to test their effectiveness in cancer therapy, with the MCF-7 cancer cell line being the most commonly used. A literature search was conducted in four major databases: PubMed, SciELO. The Boolean operator “and” was used to refine the search strategy, combining the terms Schiff base, breast cancer, MCF-7 and metal complexes. Studies published between 2020 and 2025 investigating the cytotoxic activity of metal complexes with Schiff base ligands on the MCF-7 breast cancer cell line were included in the analysis. Articles were considered eligible if they were written in English. As a result of the database search, 37 scientific articles were selected and divided into three groups based on the ligand structure. The largest group of articles described the synthesis, structure, and anticancer activity of metal complexes with ligands based on the salicylaldehyde structure. These were included in the first group of complexes described. The second, extremely interesting and promising group of compounds consisted of metal complexes with ligands containing a sulfur atom. The last group included metal complexes with Schiff base ligands that were not included in the two previously mentioned groups. As indicated by the research results contained in the reviewed articles, Schiff base metal complexes constitute an interesting group of compounds characterized by a range of activities, including anticancer activity, which may in the future be used in anticancer therapy. They may also represent a cheaper and more effective alternative to platinum-based drugs.

## 1. Introduction

Cancer is a disease in which some cells begin to grow uncontrollably and spread to other parts of the body. Cancer can begin in any of a large number of cells in any part of the body. According to WHO data, breast cancer is the most common cancer worldwide. In 2022, 2,296,840 cases of breast cancer were reported, 670,000 of which resulted in death. This cancer occurs in all countries and primarily affects women. In men, it affects only 0.5–1.0% of the population [1]. It has been known for some time that breast cancer is not a single disease, but rather a series of molecularly distinct tumors that develop from breast epithelial cells [2].

Various cell lines play a key role in cancer research. They must be capable of being used in a wide range of studies, particularly in vitro cancer models. The MCF-7 cancer cell line has been widely used in many research centers for over 40 years. It was isolated in 1973 from a 69-year-old patient at the Michigan Cancer Foundation (the first letters form the abbreviation of the cell line name) [3]. Currently, due to its widespread use, the MCF-7 cell line has provided more data on patient care than any other breast cancer cell line. This led us to select it as the primary breast cancer cell line in our literature review.

**Figure 1:**
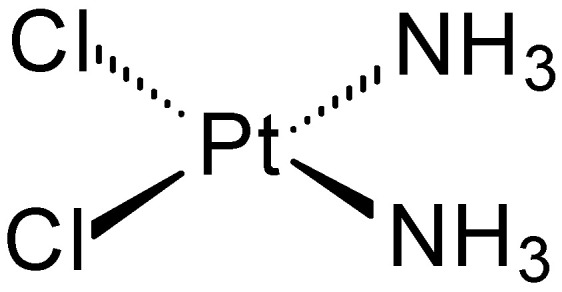
Structure of cisplatin.

Many compounds containing metal ions exhibit interesting anticancer effects. One of the first compounds with documented anticancer activity was cisplatin (*cis-*diamminedichloroplatinum(II)) (Figure 1), discovered in 1844 [4]. Its biological activity was discovered in 1965 by Barnett Rosenberg [5] and revolutionized cancer chemotherapy in humans. It was approved for medical use by the FDA in 1978 [6,7]. To this day, it is often the reference compound in comparisons of anticancer activity.

Their action involves covalent metal binding to DNA and forming cross-links, which disrupt DNA replication and transcription, resulting in cell death [8,9,10,11]. Other mechanisms of action of metal complexes include the ability to generate reactive oxygen species (ROS), e.g., arsenic trioxide, which can cause DNA impairment, mitochondrial dysfunction, and ultimately cell death [12,13]. Furthermore, platinum and gold compounds may exhibit antiangiogenic properties, which can inhibit tumor growth and spread by disrupting blood supply [14]. Although metal complexes often exhibit compelling anticancer properties, their clinical application often faces significant challenges related to toxicity, resistance, and selectivity. This presents researchers with the challenge of overcoming these barriers for more effective and targeted treatment. One approach may be to utilize specific ligands in metal complexes, such as Schiff bases.

Schiff bases, also known as imines, were discovered by the German chemist Hugo Schiff and are defined as chemical compounds (imines) bearing a hydrocarbyl group on the nitrogen atom R_2_C = NR′ (R′ ≠ H). They are considered by many to be synonymous with azomethines [15]. Schiff bases of many metals, including V, Mn, Co, Fe, Ni, Cu, Zn, Ru, Ir, and various lanthanides, may be promising compounds in anticancer therapy [16,17,18,19,20,21,22,23]. Their anticancer properties result, among others, from their ability to bind to nuclear DNA, mitochondrial DNA, and G-quadruplex DNA [24,25,26,27]. It is worth emphasizing that complexes of non-noble metal ions with Schiff bases can be used as effective agents in overcoming multidrug resistance (MDR) via several mechanisms of action [28].

## 2. Materials and Methods

This literature review focuses on the synthesis and cytotoxic activity of metal Schiff base complexes. The complexes presented in this publication were characterized using various analytical and spectral methods. The structures of the compounds were determined based on X-ray diffraction, elemental and spectral analysis, including IR, UV–Vis, ^1^H-NMR, mass spectra, electron spin resonance (ESR), magnetic moments, molar conductivity, thermal analysis and electron microscopy.

A literature search was conducted in four major databases: PubMed and SciELO. The Boolean operator “and” was used to refine the search strategy, combining the terms Schiff base, breast cancer, MCF-7 and metal complexes. Studies published between 2020 and 2025 investigating the cytotoxic activity of metal complexes with Schiff base ligands on the MCF-7 breast cancer cell line were included in the analysis. Articles were considered eligible if they were written in English.

From the initial pool of records, 37 full-text articles were ultimately selected for analysis. The studies included studies of the synthesis and cytotoxic activity of compounds against the MCF-7 cell line.

## 3. Results

### 3.1. Metal Complexes with Schiff Bases Derived from Salicylaldehyde

One of the most used methods for preparing Schiff bases is the reaction of carbonyl compounds such as aldehydes and ketones with primary amines. Salicylic aldehyde and its derivatives are frequently used substrates for the synthesis of Schiff base type ligands, and the literature contains extensive material on them [29,30,31,32,33,34,35,36,37]. Due to this wealth of information, we decided to distinguish them as a separate group.

**Figure 2:**
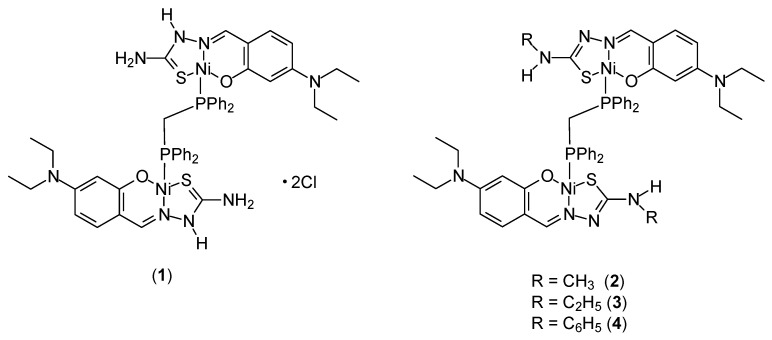
Structure of binuclear nickel(II) complexes.

Four new dinuclear Ni(II) complexes [Ni_2_(H-DEAsal-tsc)_2_(μ-dppm)]·2Cl (**1**), [Ni_2_(DEAsal-mtsc)_2_(μ-dppm)] (**2**), [Ni_2_(DEAsal-etsc)_2_(μ-dppm)] (**3**) and [Ni_2_(DEAsal-ptsc)_2_(μ-dppm)] (**4**) (Figure 2) were synthesized by Kalaiarasi et al. [38]. They used ligands whose synthesis had been previously described in the literature: 4(N,N)-diethylaminosalicylaldehyde-4(N)-thiosemicarbazone [H_2_-DEAsal-tsc], H_2_L^1^/4(N,N)-diethylaminosalicylaldehyde-4(N)-methylthiosemicarbazone [H_2_-DEAsal-mtsc], H_2_L^2^/4(N,N)-diethylaminosalicylaldehyde-4(N)-ethylthiosemicarbazone [H_2_-DEAsal-etsc], H_2_L^3^/4(N,N)diethylaminosalicylaldehyde-4(N)-phenylthiosemicarbazone [H_2_-DEAsal-ptsc] and H_2_L^4^ and 1,1′-bis(diphenylphosphino)methane (dppm). The cytotoxic nature of the complexes was demonstrated on the A549 and HeLa cell lines. Significant cytotoxicity was confirmed on the MCF-7 cell line. The IC_50_ values for free ligands (in the range of 14.3–15.49 μM) were similar to those of cisplatin (16.79 ± 0.08 μM). For the complexes, these values are approximately three times lower. For Ni(II) [Ni_2_(H-DEAsal-tsc)_2_(μ-dppm)]·2Cl, [Ni_2_(DEAsal-mtsc)_2_(μ-dppm)], [Ni_2_(DEAsal-etsc)_2_(μ-dppm)] and [Ni_2_(DEAsal-ptsc)_2_(μ-dppm)] were: 5.37 ± 0.21, 4.91 ± 0.18, 4.66 ± 0.22, 5.69 ± 0.17 μM, respectively. They confirmed the intercalation interaction of the complexes with DNA based on studies using ethidium bromide (EB) and analysis of the viscosity of the DNA solution. The effectiveness of the complexes on the previously described cancer cells was further confirmed by assays of LDH release and NO production.

**Figure 3:**
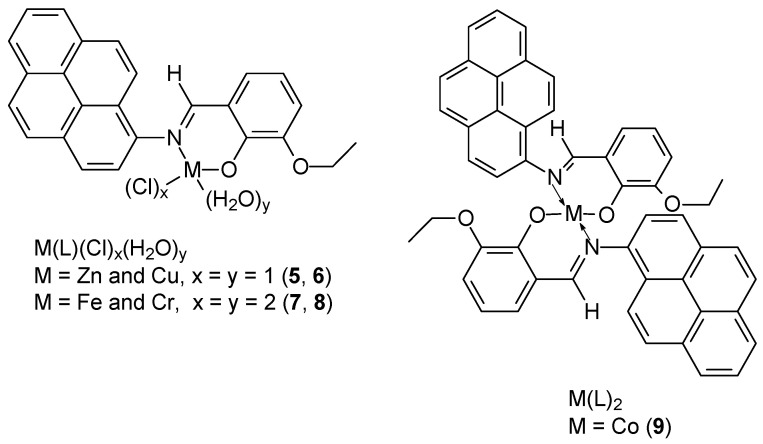
Proposed structures for metal complexes synthesized by Aazam and coworkers [39].

Promising results of cytotoxicity efficacy against MCF-7 breast cancer cells were demonstrated by newly synthesized Schiff base ((E)-2-ethoxy-6((pyren-1-ylimino)methyl)phenol) and its complexes with metals (Zn(II), Cu(II), Cr(III), and Fe(III)) in 1:1 ratio, for Co(II) 1:2 (Figure 3): Zn(L)Cl(H_2_O) (**5**), Cu(L)Cl(H_2_O) (**6**), Fe(L)Cl_2_(H_2_O)_2_ (**7**), Cr(L)Cl_2_(H_2_O)_2_ (**8**), Co(L)_2_ (**9**). NMR and IR studies showed that the formation of the Schiff base-metal complex occurs via the nitrogen atom of azomethine and the oxygen atom of the hydroxyl group of 3-ethoxy salicylaldehyde [39]. X-Ray structure studies of the complexes showed that the Cr(III) and Fe(III) complexes have distorted octahedral geometry, while for Zn(II) and Co(II) tetrahedral geometry was found. Furthermore, ESR studies indicated distorted geometry for the Cu(II) complex. Cytotoxicity studies of the compounds showed that the [Cu(II)(L)(Cl)(H_2_O)] complex (**6**) exhibited the strongest cytotoxic effect on MCF-7 cancer cells, with an IC_50_ value of 5.661 ± 0.33 μg/mL. For the Zn(II), Co(II), and Fe(III) complexes, the IC_50_ ware 12.742 ± 0.73, 21.141 ± 1.21, and 16.895 ± 0.97 (μg/mL), respectively. The determined value for 5-FU and unbound ligand was 18.047 ± 1.04, and 63.901 ± 3.67 (μg/mL), respectively.

Paliwal and colleagues [40] synthesized a new Cu(II) complex **10** with salicylidene carbohydrazide as the ligand and *o*-phenanthroline as the co-ligand. They showed that the crystal lattice of the complex in the orthorhombic system was asymmetric. The complex consisted of two distinct dinuclear copper complexes containing salicylidene carbohydrazide as the main Schiff base ligand, o-phenanthroline as the co-ligand, two nitrate anions, and two water molecules. The cytotoxicity of the Cu(II) complex for MDA-MB-231 and MCF-7 monolayer cultures was 1.86 ± 0.17 μM and 2.22 ± 0.08 μM, respectively. For spheroidal cells (3D-MDA-MB-231), the IC_50_ values were 1.51 ± 0.29 μM, giving better result than cisplatin. The binding constant of the complex to ctDNA in the ctDNA intercalation mode was K_b_ 1.25 × 10^4^ M^−1^. This allows for efficient DNA damage by both oxidative and hydrolytic pathways, leading to cell apoptosis, as indicated by Western blot results.

**Figure 4:**
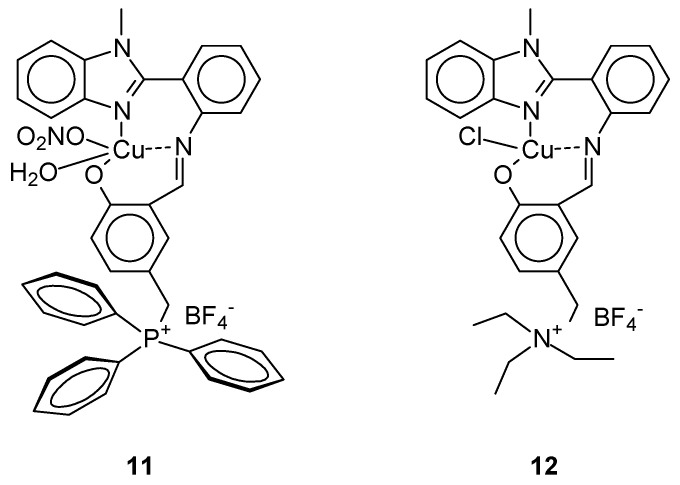
Structure of complex proposed by Paul et al. [41].

Paul et al. [41] obtained complexes that showed significant cytotoxicity not only against breast cancer cells (MCF-7) but also against human cancer cell lines A-549 (lung cancer) and HeLa (cervical cancer). The use of as Schiff base ligands in the presence of copper(II) salts (Cu(NO_3_)_2_ 3H_2_O or CuCl_2_ 2H_2_O) and in the presence of tetrafluoroborate led to obtain of two new complexes (Figure 4) [Cu(5-CH_2_PPh_3_-2-salmethylben)(NO_3_) (H_2_O)][BF_4_] 2/3(H_2_O) 1/3(MeOH) (**11**) and [Cu(5-CH_2_NEt_3_-2-salmethylben)(Cl)][BF_4_] (**12**) [41]. Cytotoxicity IC_50_ against tested cell lines MCF-7 for complex **11** and **12** was, 80.12 ± 0.016 μM, respectively. For **11** it was better than cisplatin 45.38 ± 0.92 μM.

The antiproliferative activity of the compound [Cu(5-CH_2_PPh_3_-2-salmethylben)(NO_3_) (H_2_O)][BF_4_]·2/3(H_2_O)·1/3(MeOH) (**11**) was confirmed by, among others, morphological assessment using AO/EB, detection of apoptosis induction using Hoechst/PI double staining, quantitative analysis of apoptotic cells, DNA degradation, generation of reactive oxygen species (ROS), and induction of apoptosis by mitochondrial staining.

**Figure 5:**
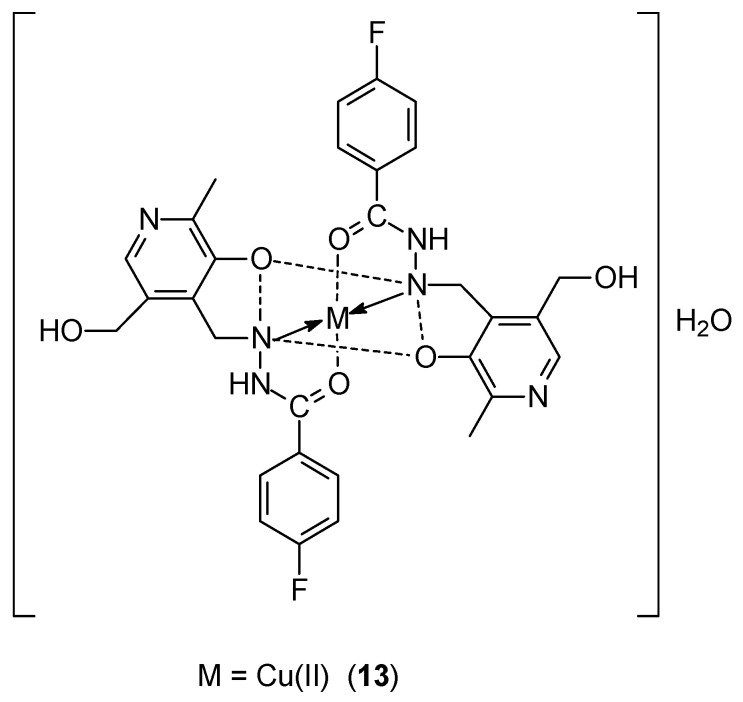
Structure of Ni(II)-PLFBH and Cu(II)-PLFBH (M = Ni(II) or Cu(II)) complexes [42].

Kavitha et al. [42], using the condensation reaction of pyridoxal and 4-fluorobenzohydrazide, synthesized a new Schiff base ligand, 4-fluoro-N-((3-hydroxy-5-(hydroxymethyl)-2-methylpyridin-4-yl)methylene)benzohydrazide (PLFBH) (Figure 5). PLFBH was complexed with Ni(II), Cu(II) **13** and Zn(II) ions. The obtained complexes exhibited octahedral geometry with hexacoordinate distortion. DNA docking studies confirmed the intercalary mode of binding of the complexes to ctDNA. These complexes are characterized by good ability to cleave pBR322 plasmid DNA. In the case of the MCF-7 cell line, the most effective activity was determined for the Cu(II)-PLFBH complex, IC_50_ was 15.3 ± 0.55 μM.

**Figure 6:**
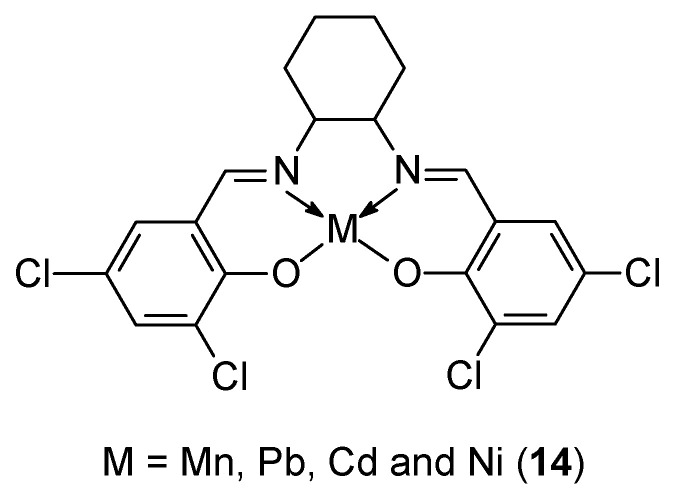
Metal complexes synthesized by Priya et al. [43].

Priya et al. [43] synthesized a new tetradentate ligand and used it to prepare four different metal complexes based on Mn(II), Ni(II), Cd(II), and Pb(II) ions (Figure 6). The ligand was synthesized based on 3,5-dichlorosalicylaldehyde and *trans*-1,2-diaminocyclohexane. Antioxidant and antibacterial properties were determined for these compounds using AutoDock Viva software, v1.1.2, demonstrating the effectiveness of the Ni(II) **14** complex in binding to BSA and DNA. A molecular docking study of the Ni(II) complex demonstrated synergistic activity between drugs and biomolecules. The compound demonstrated efficacy of 68.28% at a concentration of 320 μg/mL. The IC_50_ value was determined to be 108.1 µg/mL. This study therefore confirms the feasibility of these newly synthesized Schiff base derivative complexes as prodrugs in clinical trials.

**Figure 7:**
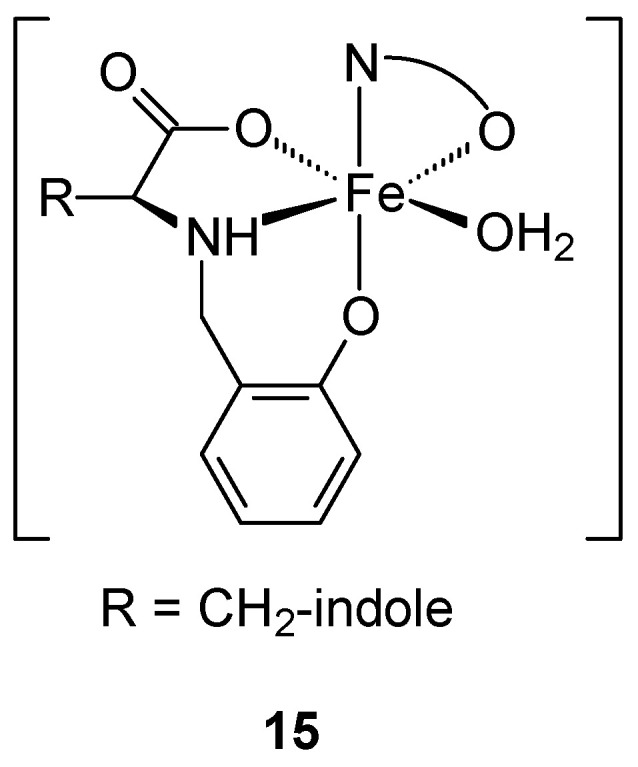
General formula of the iron(III) complex obtained by Ferretti et al. [44].

The obtained mixed-ligand Fe(III)-aminophenolate complexes (Figure 7), obtained from salicylaldehyde and L-tryptophan, with quinoline derivatives as co-ligands: 8-hydroxyquinoline (8HQ) [Fe(L)(8HQ)(H_2_O)] (**15**) and its 5-chloroderivative (Cl8HQ) [Fe(L)(Cl8HQ)(H_2_O)] (**16**) may be an alternative to commonly used anticancer drugs. Spectroscopic studies show that the obtained complexes have an octahedral geometry, with the aminophenolate acting as a tridentate dianion ligand and the 8HQ co-ligands as bidentate chelates. The obtained complexes demonstrated higher activity than cystatin. The determined IC_50_ values against MCF-7 for [Fe(L)(Cl8HQ)(H_2_O)] [Fe(L)(Cl8HQ)(H_2_O)] and cystatin were 4.3 ± 0.2 μM, 10.7 ± 2.5 μM, and 42 ± 3 μM, respectively. Despite the lower antitumor activity of complex **16** compared to complex **15**, its IC_50_ values remain significantly lower 4-fold—against cisplatin (CDDP) in the analyzed cell line. It is worth emphasizing that both complexes (**15**, **16**) demonstrate activity against the HT-29 colon adenocarcinoma line, which is resistant to CDDP. However, complex **16** shows higher selectivity, measured on a non-cancerous fibroblast cell line (L929). The selectivity index (SI) values were 8.0 and 18.8 for compounds **15** and **16** in bone cancer cells, respectively [44].

**Figure 8:**
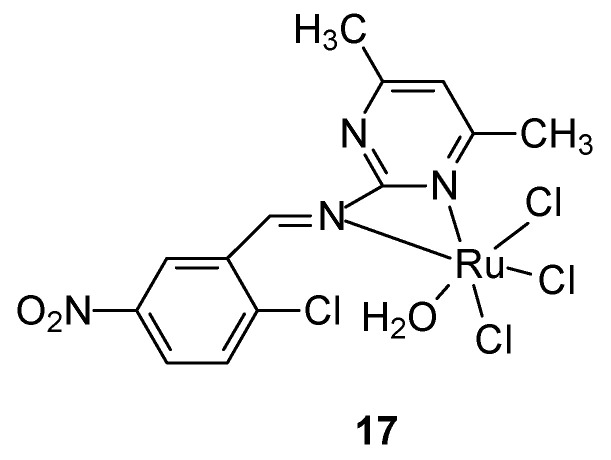
Ruthenium complex proposed by Noureldeen et al. [45].

For the synthesis of a new ruthenium-pyrimidine complex **17** (Figure 8), a Schiff base was obtained by the reaction of 2-amino-4,6-dimethylpyrimidine and 2-chloro-5-nitrobenzaldehyde in ethanolic solution, obtaining a yellowish-white solid product, which was complexed with a metal salt—RuCl_3_. It effectively inhibited the tested cancer cells, the IC_50_ value for breast cancer cells T47D was 38.5 μM and 46.7 μM for MCF-7 cells, respectively [45].

**Figure 9:**
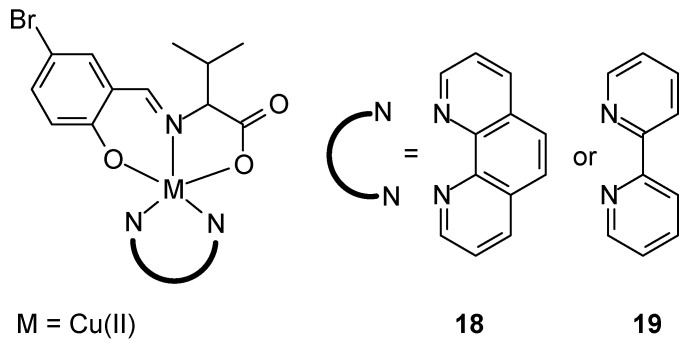
Cu(II) complexes with tridentate ligand (HL) and bidentate diamines (1,10-phenanthroline or 2,2′-bipyridyl) [46].

New ternary metal complexes of Co(II), Ni(II), Cu(II), and Zn(II) ions were synthesized by reacting appropriate metal salts with amino acid Schiff bases in the presence of heterocyclic bases (Figure 9), but only Cu(II) complexes was tested against antitumor activity. These complexes were found to possess a distorted, square pyramidal geometry around the metal ions. Cytotoxicity studies of Cu(II) complexes demonstrated their anticancer efficacy. In vitro cytotoxicity of the [Cu(L)(phen)] complex **18** demonstrated promising anticancer activity against MCF-7 cancer cell lines (17.13 ± 0.74 µM), when [Cu(L)(bpy)] (**19**) 33.18 ± 1.14 µM. Additionally, promising IC_50_ results were obtained for A549 lung cancer cells (25.95 ± 1.82 µM) and HeLa cervical cancer cells (26.26 ± 1.06 µM), compared to cisplatin, for which the IC_50_ was 17.91 ± 0.12 µM and 16.13 ± 0.16 µM, respectively [46].

**Figure 10:**
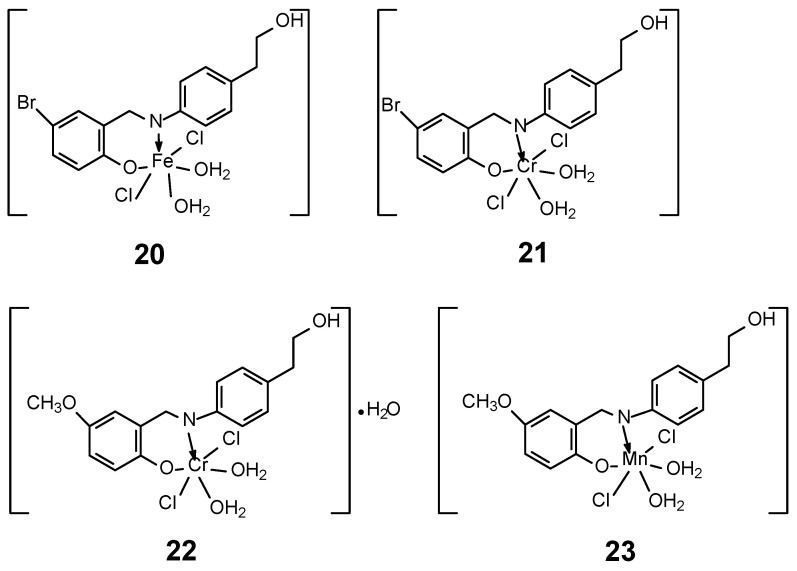
Four complexes of Mn(II), Fe(III) and Cr(III) with two Schiff base ligands synthesized by Abdel-Rahman et al. [47].

In 2023, Abdel-Rahman et al. [47] developed the synthesis of four new complexes of Mn(II), Fe(III) and Cr(III) with two Schiff base ligands with the general formulas [M(L)Cl_2_(H_2_O)_2_] for FeL1 **20**, CrL1 **21** and CrL2 **22** and [M(L)Cl(H_2_O)_3_] for MnL2 **23** (Figure 10). Ligands (L) were obtained in the synthesis of an ethanol solution of 2-(4-aminophenyl)ethan-1-ol with 5-methoxy salicylaldehyde or 5-bromo salicylaldehyde, obtaining 4-bromo-2-[(*E*)-{[4-(2-hydroxyethyl)phenyl]imino}methyl]phenol and 2-[(*E*)-{[4-(2-hydroxyethyl)phenyl]imino}methyl]-4-methoxyphenol. The geometry of the complexes adopted an octahedral structure, confirmed by DFT/B3LYP calculations using the Gaussian 09 program, as well as UV-vis spectra and magnetic moment measurements. Furthermore, the MTT technique was used to demonstrate antibacterial and antifungal properties. The cytotoxicity of the obtained complexes was also confirmed. The highest activity was observed for the Mn(L2)(H_2_O)_3_Cl] compound, with an IC_50_ value of 3.0 ± 0.2 μg/mL in relation to the cell line MCF-7. Additionally, a low value was observed against Hep-G2 (2.6 ± 0.11 μg/mL). For comparison, the value for cystatin was 4.0 μg/mL for MCF-7 cell line. The studies of the obtained complexes were extended to determine the affinity of the compounds to bind and the ability to cleave ctDNA, using UV-vis and gel electrophoresis. The binding affinity of the new complexes for DNA was in the series FeL1 > MnL2 > CrL2 > CrL1 and amounts to K_b_ 10 × 10^4^ for the Fe(III) complex, while for CrL1 it was 2.1 × 10^4^. The determined binding energy of the ligands and their complexes indicates a strong affinity for the PDB:1bna receptor, in the order FeL1 > MnL2 > CrL2 > CrL1 > HL1 > HL2. The binding energy for FeL1 was −22.0 kcal/mol [47].

**Figure 11:**
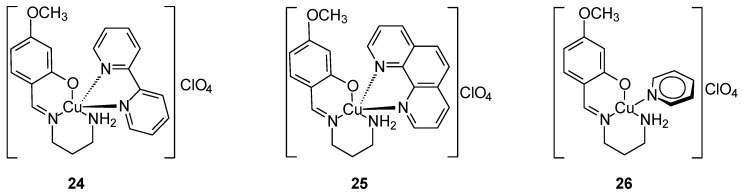
Three complexes of Cu(II) synthesized by Ghasemi [48].

Strong anticancer activity was demonstrated by new Cu(II) complexes with a mixed ligand [Cu(SB)L1–3]ClO_4_ (Figure 11) where SB is the 1:1 condensation product of 1,3-propanediamine with 2-hydroxy-4-methoxybenzaldehyde, which was formed during the synthesis of the matrix of complex. The complex contains a tridentate asymmetric NN’O-type Schiff base ligand (SB) and a heterocyclic co-ligand (L): 2,2′-bipyridine (L2, **24**), 1,10-phenantroline (L3, **25**) and pyridine (L1, **26**). Complexes **25** and **26** were formed by replacing the monodentate py with the bidentate bpy or phen, respectively. In addition to strong anticancer activity against the MCF-7 cell line presenting in order **25** < **24** < **26**. After 48 h of incubation the IC_50_ value for **25** against MCF-7 was 4.97 μM. These compounds also exhibited cytotoxic activity against colon (HCT116) and lung (A549) cancer cells [48].

**Figure 12:**
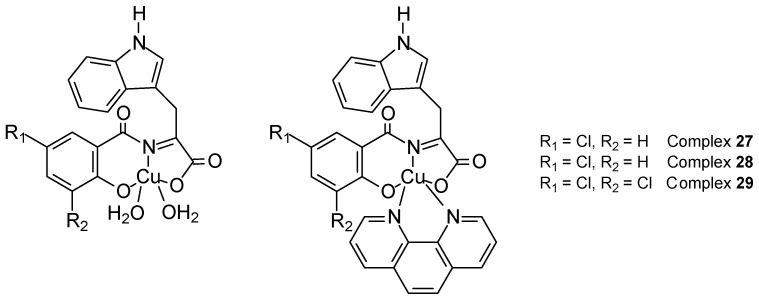
Three new copper(II) complexes with Schiff base derived from 5-chlorosalicylaldehyde and L-tryptophan [49].

Gültekin et al. [49] synthesized three new copper(II) Schiff base complexes (Figure 12) obtained from chlorosalicylaldehyde and L-tryptophan: [Cu(5-ClSal-Trp)(H_2_O)_2_] (**27**), [Cu(5-ClSal-Trp)(phen)]⋅C_2_H_5_OH (**28**), and [Cu(3,5-ClSal-Trp)(phen)] (**29**) (5-ClSal-Trp). Their crystal structure exhibited pyramidal coordination geometry. Interactions of ctDNA and BSA complexes showed moderate intercalation. Viability assay with sulforhodamine B (SRB) demonstrated cytotoxicity on breast cancer cell lines (MCF-7 and MDA-MB-231) and healthy breast epithelial cells (MCF-10A). The most effective apoptosis inducer was the [Cu(3,5-ClSal-Trp)(phen)] complex, which was confirmed by fluorescent staining. After 72 h, the IC_50_ value for **29** was 4.31 ± 1.1 µM.

**Figure 13:**
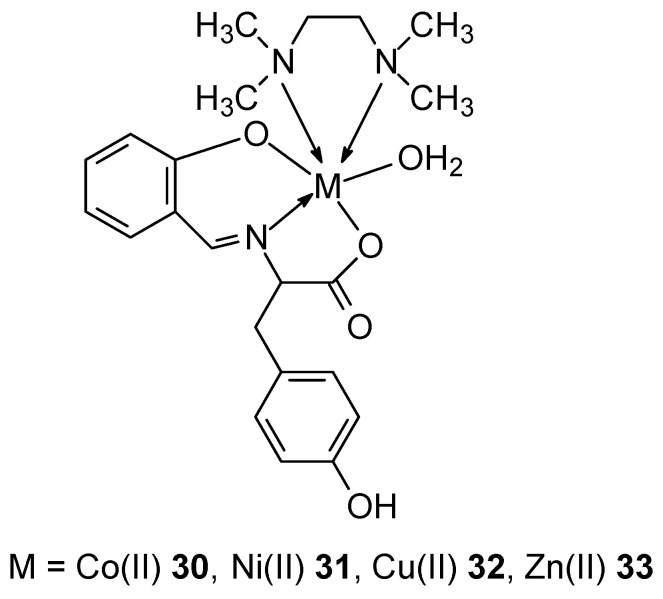
Complexes of Co(II), Ni(II), Zn(II), and Cu(II) with Schiff bases derived from L-tyrosine and salicylaldehyde [50].

Complexes **30**–**33** containing the divalent metal ions Co(II), Ni(II), Zn(II), and Cu(II) were synthesized using Schiff bases derived from L-tyrosine and salicylaldehyde (Figure 13) [50]. Using electron absorption titration, the interaction of the metal complexes with ctDNA was demonstrated, with the Cu(II) complex **33** exhibiting the highest intrinsic binding constant (K_b_) of 3.46 ± 0.02 × 10^5^ M^−1^. Cytotoxicity studies using the MTT assay also demonstrated that the copper complex exhibits the highest cytotoxicity even at very low concentrations. These properties of the complex can be attributed to chelation, which enhances the lipophilic nature of the complexes and thus penetration through the lipid layer of the cell membrane.

**Figure 14:**
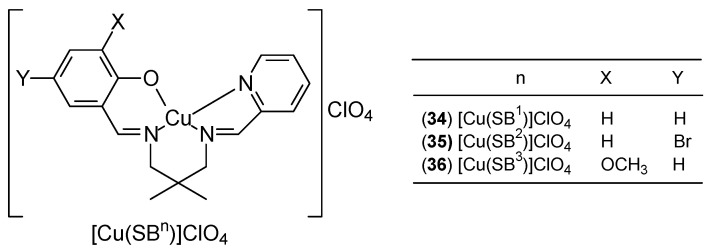
Schematic structure of three new Cu(II) Schiff base complexes [Cu(SB_n_)]ClO_4_ [51].

Three new Cu(II) Schiff base complexes [Cu(SB_n_)]ClO_4_, (where n = 1 for **34**, = 2 for **35** and = 3 for **36**) represent a new therapeutic potential (Figure 14). The unsymmetrical N_3_O-type ligands, belonging to the Schiff base group (SB_1–3_), were obtained by stepwise condensation of one equivalent of 2,2-dimethyl-1,3-propanediamine with one equivalent of three different aldehydes: salicylaldehyde, 5-bromosalicylaldehyde, or 3-methoxysalicylaldehyde (for complexes with n = 1, 2, 3, respectively). The second component in all cases was pyridine-2-carbaldehyde. Studies on three human cancer cell lines: colon (HCT116), lung (A549), and breast (MCF-7) have shown that the complexes have good cytotoxic activity, with in vitro studies for MCF-7 demonstrating the following order of antiproliferative activity: [Cu(SB_3_)]ClO_4_ > [Cu(SB_2_)]ClO_4_ > [Cu(SB_1_)]ClO_4_. However, the effect of substituents in the complexes on antiproliferative activity has not been determined [51].

**Figure 15:**
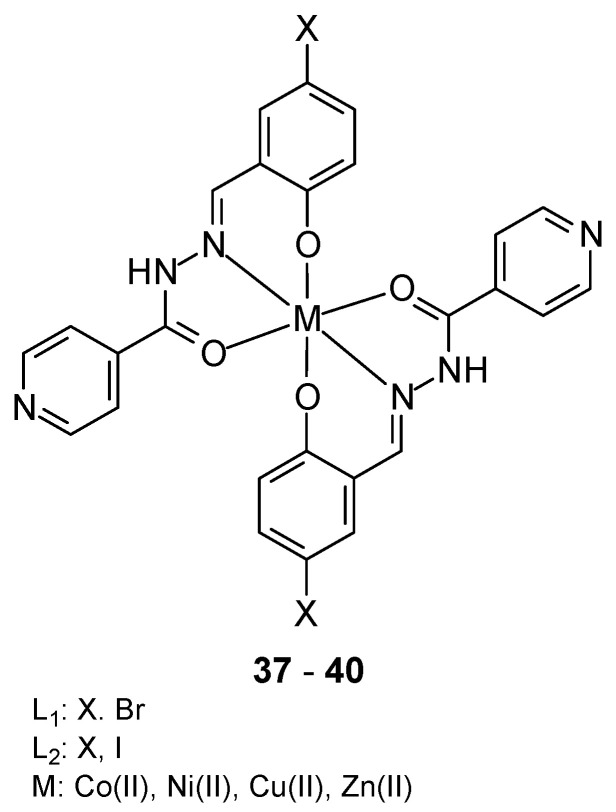
Complexes based on Co(II), Ni(II), Cu(II), and Zn(II) ions with (E)-N′-(2-hydroxy-5-iodobenzylidene)isonicotinohydrazide as a Schifft base [52].

Mononuclear metal complexes **37**–**40** based on Co(II), Ni(II), Cu(II), and Zn(II) ions, isoniazid, benzaldehyde (L1) and 5-halo-2-hydroxy-benzaldehyde (L2) (Figure 15) were synthesized by Devraye et al. [52]. The O and N donor atoms from the Schiff base ligands coordinated the metal ions in an octahedral geometry. In vitro cytotoxicity studies against the human cancer cell line MCF-7 showed that the IC_50_ values for the complexes [Co(L1)_2_], [Ni(L1)_2_], [Cu(L1)_2_] and [Zn(L1)_2_] were 7.26, 70.93, 128.32 and 2.73 μg/mL, respectively, while for 5-flurouracil (13.38 μg/mL). The obtained complexes exhibited strong inhibitory activity against the MCF-7 cancer cell line.

Table 1 summarizes the information contained in this chapter.

### 3.2. Examples of Metal Complexes with Sulfur-Containing Schiff Bases

Carbothioamide compounds offer potential for the design of anticancer drugs. The presence of an azomethine group in the macrocyclic chelate ring plays a key role in the ligand activity of these compounds [53,54]. Ternary metal complexes represent a new perspective in the synthesis of potential anticancer drugs.

**Figure 16:**
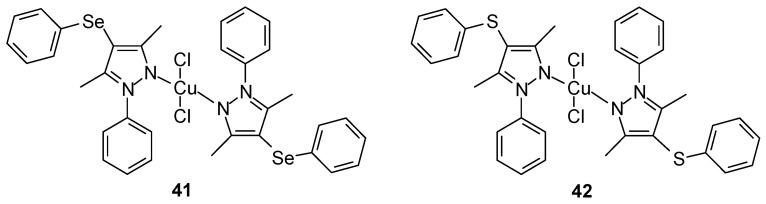
Cu(II) complexes based on the monodentate bis(ligand) 4-(arylchalcogenyl)-1H-pyrazole containing selenium and sulfur group [56].

Cu(II) complexes **41**–**42** based on the monodentate bis(ligand) 4-(arylchalcogenyl)-1H-pyrazole containing selenium (proligands: [3,5-dimethyl-1-phenyl-4-(phenylselanyl)-1H-pyr-azole] [55]) or sulfur (proligands: [3,5-dimethyl-1-phenyl-4-(phenylsulfur)-1H-pyrazole]) (Figure 16) were synthesized by Pinheiro et al. [56].

Free ligands alone did not demonstrate antitumor activity. Studies with the selenium-containing complex demonstrated good activity against the MCF-7 cell line (IC_50_ 44 ± 11 μM), but it was characterized by low selectivity (selectivity index 1.12–1.40). The sulfur-containing complex was found to be selective for MCF-7 with an IC_50_ of 59 ± 2 μM.

**Figure 17:**
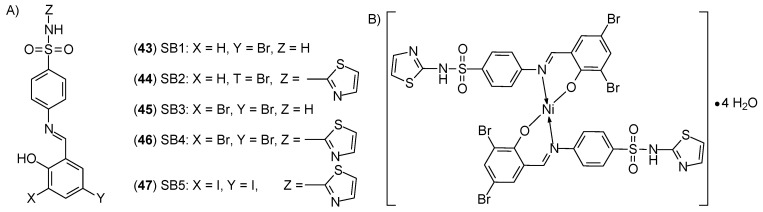
Structure of: (**A**) sulfonamide-Schiff base ligands (SB^1^–SB^5^), (**B**) Ni(SB^4^–H)_2_]·4H_2_O complex [57].

Elsamra and al. [57] synthesized sulfonamide-based nickel(II) ion complexes SB^1^-SB^5^ (**43**–**47**) (Figure 17) represent a new type of compound with anticancer properties. They differ from each other by a substituent in the form of a halogen atom (bromine or iodine) in the part derived from salicylaldehyde. The highest activity was determined for the [Ni(SB^4^-H)_2_]·4H_2_O complex, which may be related to the presence of two bromine atoms at the 3- and/or 5-positions of the phenolic ring and the thiazole ring. The IC_50_ value for this complex was 4.33 ± 0.5 μM, while the value determined for cisplatin was 19.0 ± 2.3 μM, suggesting a four-fold increase in the efficacy of the tested complex. For comparison, the IC_50_ value for the complex [Ni(SB^5^–H)(OH)·(H_2_O)], in which the iodine atom was located at the 3- and 5-position of the phenol group, was >100 μM. It seems that the [Ni(SB^4^-H)_2_]·4H_2_O complex may be an effective alternative to cisplatin.

**Figure 18:**
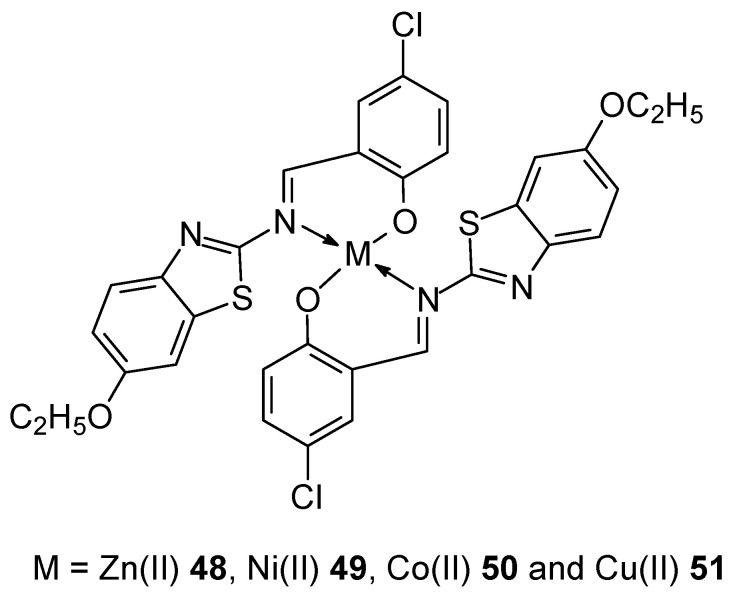
Structure new complexes prepared by Gopichand et al. [58].

2-((*E*)-(6-ethoxybenzo[d]thiazol-2-ylimino)methyl)-4-chlorophenol (2-EBTMCP) is an example of a new Schiff base (HL) used for complexation of divalent metal ions with anticancer potential (Figure 18). It was obtained by the reaction of 6-ethoxy-2-aminobenzothiazole and 5-chloro-2-hydroxybenzaldehyde. As a result of the complexation, compounds with a stoichiometric ratio of 1:2 were obtained. They demonstrate the ability to bind to DNA. The in vitro MTT assay showed that the order of IC_50_ values of the metal complexes for HeLa and MCF-7 cell lines was: Zn(II) **48** > Ni(II) **49** > Co(II) **50** > Cu(II) **51** > Ligand [58].

**Figure 19:**
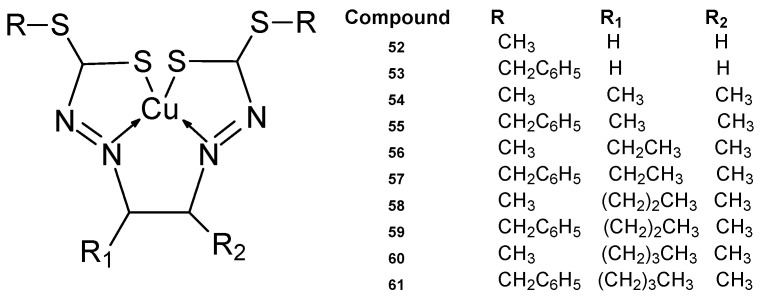
Series of copper ion complexes **52**–**61** developed by Break et al. [63].

Schiff bases whose ligand structure is based on dithiocarbazanes having a chelating nitrogen atom and sulfur with complexing properties exhibit a wide range of biological activity [59,60,61], which is enhanced by chelating with many metal ions, especially transition metals [62]. A new series of copper ion complexes developed by Break et al. [63] (Figure 19) was tested for anticancer activity against MCF-7 and MDA-MB-231 cells. They were synthesized by reacting selected dicarbonyls with S-methyldithiocarbazate (SMDTC) and S-benzyldithiocarbazate (SBDTC) and finally complexing them with copper(II) ions [63,64]. From the new macroacyclic Cu(II) complexes, the Cu1 (**52**) complex demonstrated the highest cytotoxic activity against MCF-7, at 1.7 ± 0.1 µM. The SMDTC-glyoxal combination demonstrated a better activity of 1.4 µM. The Cu4 **55**, Cu5 **56**, and Cu8 **59** complexes had good IC_50_ values of 11 ± 1.9 µM, 14 ± 2.1 µM, 7.3 ± 14 µM compared to cisplatin, which had an IC_50_ of 25 ± 0.3 µM. It turned out that there was no relationship between chain length and cytotoxic activity. For free ligands, cytotoxic activity ranged from 2.6 to 22 µM.

**Figure 20:**
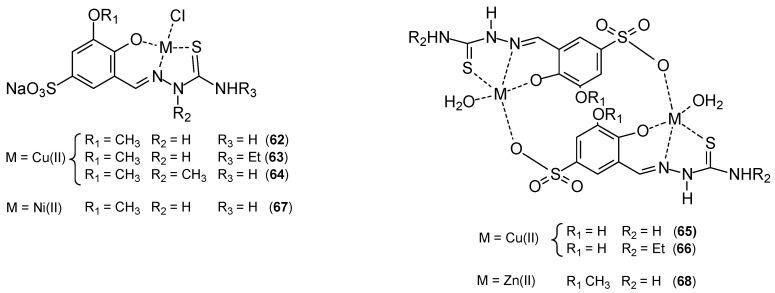
Complexes **62**–**68** prepared by Miglioli’s et al. [65].

Copper complexes exhibit promising anticancer properties, but often exhibit poor aqueous solubility, which significantly limits their potential use in the pharmaceutical industry. Miglioli’s research group developed the synthesis of copper(II) complexes with salicylaldehyde thiosemicarbazone ligands [65,66,67] (Figure 20). These compounds exhibited nanomolar cytotoxic activity but very poor solubility. To improve solubility in aqueous solutions, a sulfonic acid group was introduced into the ligand structure. 2,3-hydroxy or 2-hydroxy-3-methoxybenzaldehyde was condensed with aniline. The resulting Shiff base was then sulfonated with excess of concentrated sulfuric acid, and the product was isolated by precipitation and then hydrolyzed to yield the sulfonated aldehyde. The condensation reaction between benzaldehyde 2,3-dihydroxy-5-sulfonate or 2-hydroxy-3-methoxy-5-sulfonate and the appropriate thiosemicarbazide led to the formation of the ligands NaH_2_L^1^-NaH_2_L^5^. The ligands were complexed with CuCl_2_·2H_2_O in methanol. The copper(II) complexes exhibited high selectivity towards cancer cells. The in vitro antitumor potential of the complexes and cisplatin was assessed on the following human cancer cell lines: HCT-15 (colon), 2008 (ovarian), PSN-1 (pancreas), A431 (cervix), MCF-7, and MDA-MB-231 (breast). Cytotoxicity parameters expressed as IC_50_ and obtained after 72 h of drug exposure using the MTT assay indicated that free ligands were not effective against the tested cell lines. Promising results were obtained for copper(II) complexes. They demonstrated remarkable cytotoxicity against the exposed cancer cell lines, with mean IC_50_ values in the low/submicromolar range, ranging from 1.3 to 8.3 μM for the MCF-7 cell line, while for cisplatin it was 8.8 ± 0.2 μM.

**Figure 21:**
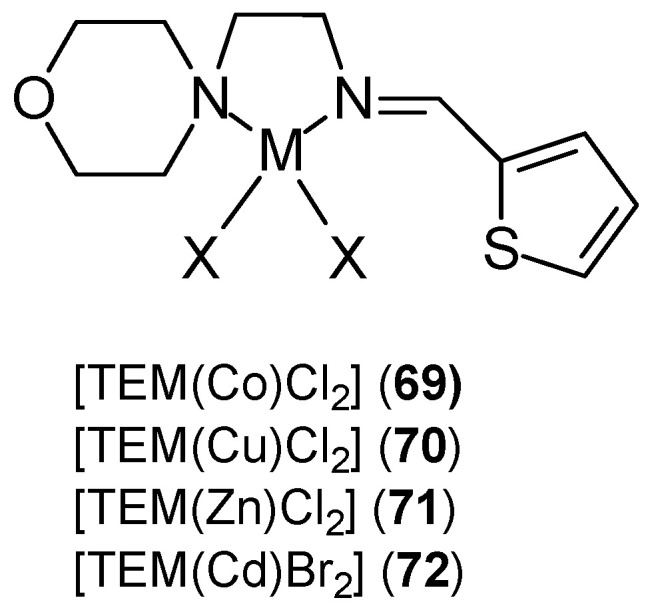
Thiophene-based metal ion complexes [68].

Nayab et al. [68] synthesized thiophene-based complexes **69**–**72** of the [TEM(M)X_2_] type (Figure 21). They synthesized them by condensing thiophene-2-carboxaldehyde with 2-morpholinoethanamine. (*E*)-2-morpholino-N-(thiophen-2-ylmethylene)ethanamine was used as a Schiff base ligand in combination with divalent metal ions (M = Co, Cu, Zn; X = Cl or M = Cd, X = Br). Analysis of the [TEM(M)X_2_] structure revealed distorted tetrahedral geometry around the M(II) center.

**Figure 22:**
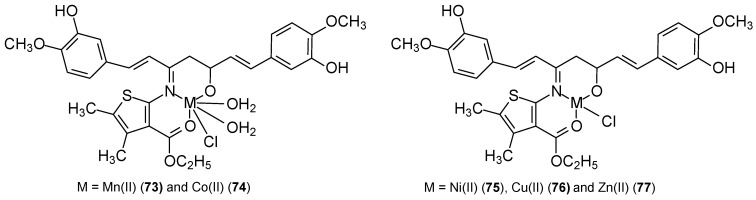
Structure of metal complexes [69].

Studies have shown that the activity of these complexes is related to the ability to bind to DNA chains and block cancer cell division. The synthesized complexes demonstrated enhanced anticancer activity against MCF-7 cells and, additionally, against the HepG2 and HCT-116 cell lines. In vitro studies of the synthesized ligand and its complexes also demonstrated antiurease and leishmanicidal properties. The cytotoxic potential of the M(II) complexes [TEM(Co)Cl_2_] (**69**), [TEM(Cu)Cl_2_] (**70**), [TEM(Zn)Cl_2_] (**71**), [TEM(Cd)Cl_2_] (**72**) and TEM was 4.0 ± 1.06 μM, 5.9 ± 0.23 μM, 3.3 ± 0.01 μM, 2.2 ± 0.09 μM, 4.0 ± 1.06 μM, and 6.0 ± 1.00 μM, respectively. The control substance was vinblastine, for which the IC_50_ was 0.98 ± 0.22 μM. The results obtained for complexes of Mn(II), Co(II), Ni(II), Cu(II), and Zn(II) ions with a ligand (CNAT) containing curcumin and a 2-aminothiophene derivative were inferior. New CNAT ligands were synthesized by refluxing an equimolar ethanolic solution of extracted curcumin and synthesized 2-amino-3-carboxyethyl-4,5-dimethylthiophene [69]. The Cu(II) complex **76** (Figure 22) demonstrated activity against MCF-7 at concentrations > 80 μg/mL, while for the remaining metal ions (**73**–**75**, **77**) this concentration was <10 μg/mL. The authors believe that this activity depends on the coordination site, the nature of the metal anion and cation, and the resulting ability to bind to DNA. According to Tweedy’s chelation theory [70], the cytotoxic effect of the complexes results from the coordination ability of the metal.

**Figure 23:**
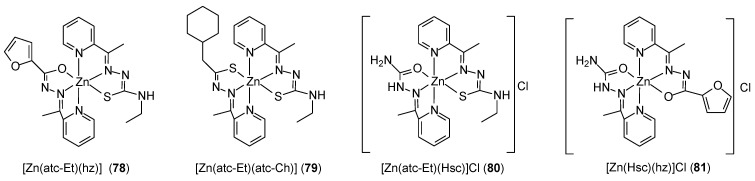
Heteroleptic Zn(II) complexes synthetized by Machado et al. [71].

Machado et al. [71] developed a method of obtaining of heteroleptic complexes of Zn(II) ions with Schiff base ligands [Zn(hz)(atc–Et)] (**78**), [Zn(atc–Et)(atc–Ch)] (**79**), [Zn(atc–Et)(Hsc)]Cl (**80**), and [Zn(hz)(Hsc)]Cl (**81**) (Figure 23). Biological studies have shown that they are active against MCF-7 cell lines (luminal BC), MDA-MB-453 (HER2-positive BC), and MDA-MB-231 (TNBC). IC_50_ values ranged from 0.01 µM for complex **79** and **80** against the MDA-MB-453 cell line to 20 µM for complex **80** against MCF7 cells. Moreover, complex **81** inhibited colony formation and migration of TNBC cells and further sensitized TBNC cells to doxorubicin and paclitaxel, most likely modulating the mechanism of epithelial–mesenchymal transition, as evidenced by increased CDH1 expression.

Alghabban et al. [72] proposed synthesis of complexes based on the ligand (*Z*)-2-((*E*)-1-(2-(4-chlorophenyl)hydrazinylidene)propan-2-ylidene)-N-phenylhydrazine-1-carbothioamide and co-ligand 1,10-phenanthroline (PHEN). They obtained complexes of Fe(III) **82**, Co(II) **83**, and Cu(II) **84** that had octahedral geometry. 1,10-Phenanthroline co-ligand forms hybrid systems in combination with Schiff base ligands. Such combinations exhibit synergistic effects, enhancing therapeutic anticancer and antimicrobial properties and increased selectivity towards cancer cells [28,73]. In particular, the copper(II) complex [Cu(CHPT)(PHEN)(H_2_O)]·2Cl·H_2_O showed higher anticancer efficacy against MCF-7 breast cancer cells (IC_50_ = 10.5 µg/mL), while the ligand (CHPT) alone (IC_50_ = 12.5 µg/mL). For the Fe(II) and Co(II) complexes, the IC_50_ ware 20 µg/mL and 23 µg/mL, respectively. The Cu(II) complex shows promising activity against K-562 leukemia cells (IC_50_ = 287.19 µg/mL).

**Figure 24:**
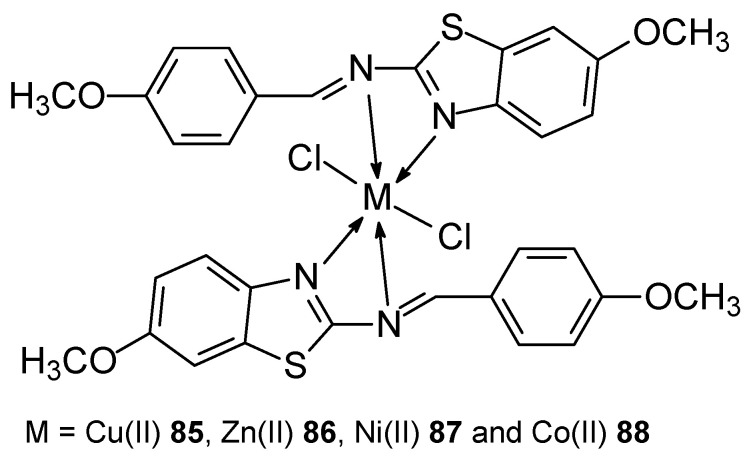
New metal(II) complexes Cu(II), Zn(II), Ni(II), Co(II) with the ligand (E)-7-methoxy-N-(4-methoxybenzylidene)benzo[d]-thiazol-2-amine.

The synthesis of new transition metal(II) complexes (Figure 24): Cu(II) **85**, Zn(II) **86**, Ni(II) **87**, Co(II) **88** with previously obtained ligand (*E*)-7-methoxy-N-(4-methoxybenzylidene)benzo[d]-thiazol-2-amine [74,75] was published by Michael et al. [76]. The Schiff base was obtained by condensation of an ethanolic solution of 2-amino-6-methoxybenzothiazole and 4-methoxybenzaldehyde (concentration ratio 1:1) [74]. The obtained ligand was mixed in a methanol solution with transition metal salts to obtain complexes in a stoichiometric metal-ligand ratio of 1:2. The IC_50_ values for the obtained complexes were higher compared to the result obtained for the reference compound, which was cisplatin. The best result was obtained for the Cu(II) complex and was 12 ± 0.03 μg.

**Figure 25:**
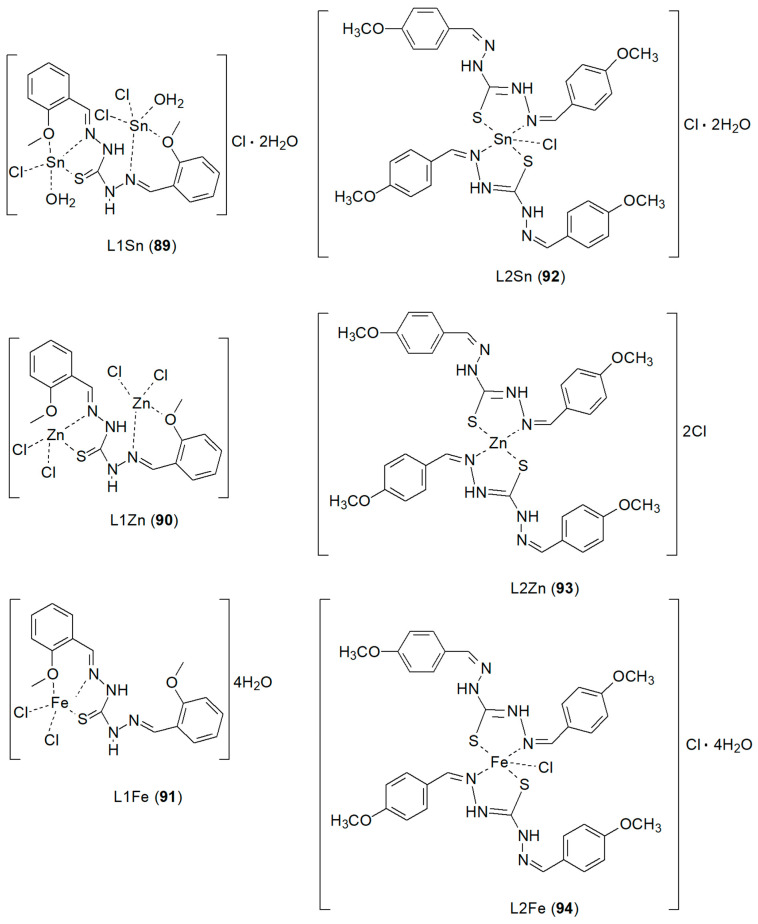
New metal complexes with Schiff base ligands prepared by condensation of thiocarbohydrazide (TCH) with *o*-anisaldehyde or *p*-anisaldehyde [78].

Mahdy et al. [77] proposed preparation of new Schiff base ligands by condensation of thiocarbohydrazide (TCH) [78] with *o*-anisaldehyde or *p*-anisaldehyde in ethanol. They were used to synthesize mono- and di-nuclear complexes with Zn(II), Sn(II) and Fe(II) ions (Figure 25) that showed variable antimicrobial activity against *Staphylococcus aureus*, *Escherichia coli* and *Candida albicans*. TCH, in addition to antibacterial and antiproliferative properties, also exhibit anticancer properties [79]. The structural diversity of their complexes with metals **89**–**94** results from the presence of O and S atoms acting as bridging sites. The synthesized complexes exhibited worse cytotoxic activity (ranging from 135 to 434 µM) than cisplatin, which was the reference compound. The best result, 47.69 ± 3.32 µM, was obtained for L1Zn. Notably, L1Fe and L1Zn proved to be the most promising complexes against colon cancer cells [77].

Table 2 summarizes the information contained in this chapter.

### 3.3. Metal Complexes with Other Types of Schiff Bases

**Figure 26:**
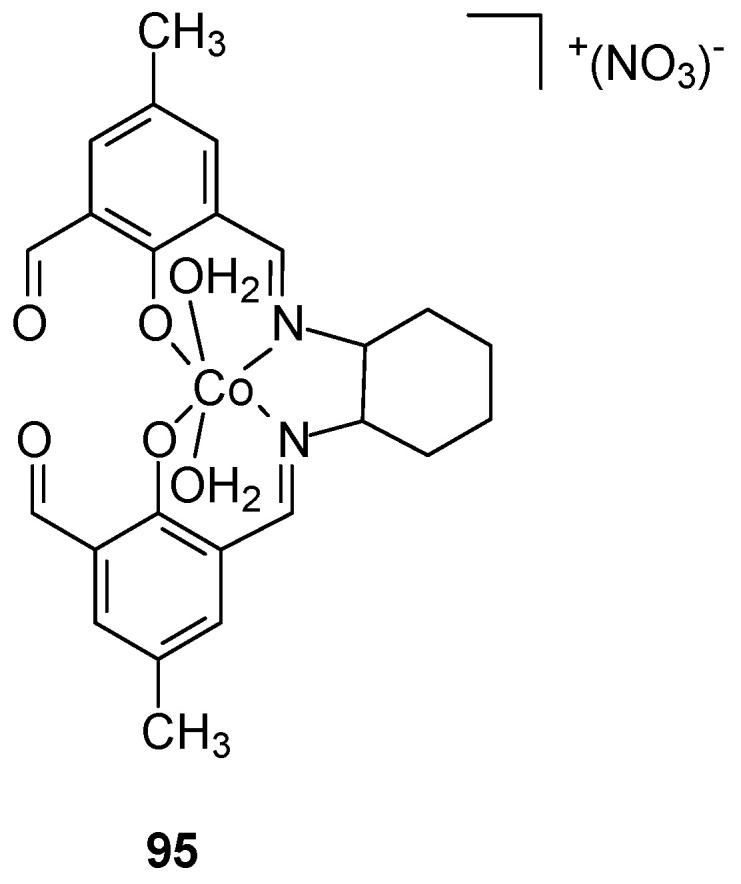
Structure of Co(III) complex **95** obtained by Dasgupta [80].

A novel mononuclear side-off compartmental complex of cobalt(III) with a Schiff base (Figure 26) was synthesized in situ by Dasgupta et al. [80]. Cyclohexane-1,2-diamine, 2,6-diformyl-4-methylphenol, and Co(NO_3_)_2_⋅6H_2_O were synthesized, yielding a complex with purity > 99%.

Cell viability testing using the MTT assay showed that the cobalt(III) complex was hemotoxic to MCF-7 cells with an IC_50_ of 16.81 ± 1.33 μM, whereas the IC_50_ for oxaliplatin was 31.4 ± 0.69 μM. Its effect was to induce apoptosis via G2-M cell cycle arrest. Furthermore, 24 h treatment did not demonstrate excessive toxicity to human PBMCs (IC_50_ ≥ 60 μM). Furthermore, in vivo studies did not reveal significant hematological, nephrotoxic, or hepatotoxicity.

**Figure 27:**
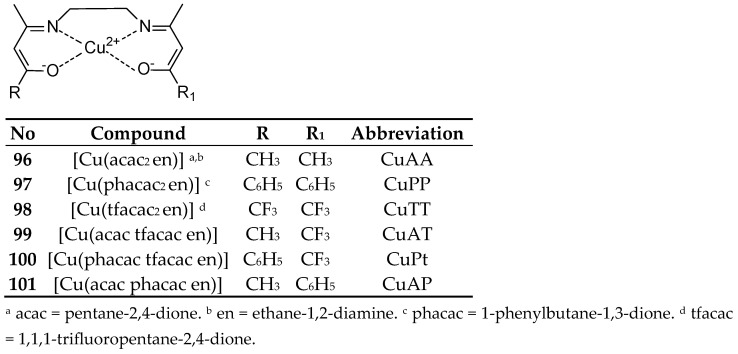
Structure of Cu(II) complex obtained by Mijatović [81].

Mijatović, and coworkers [81] synthesized Cu(II) complexes with Schiff base ethylenediamine-bis-acetylacetonate CuAA (**96**) and its derivatives in which two methyl groups were replaced by phenyl groups CuPP (**97**), CF_3_ CuTT (**98**) or mixed CH_3_/CF_3_ CuAT (**99**), Ph/CF_3_ CuPT (**100**) and Ph/CH_3_ CuAP (**101**) (Figure 27). Their cytotoxic activity showed average values of 17.53–31.40 µM against MCF-7 cancer cells. In the MTT assay, the CuAA complex demonstrated a lower IC_50_ concentration than cisplatin within 48 h, reaching values of 22.45 ± 2.27 µM and 24.7 ± 1.39 µM, respectively. However, after 72 h, cisplatin achieved a more favorable result of 7.07 ± 0.65 µM. Furthermore, CuAA demonstrated greater antioxidant properties than Trolox in the oxygen radical scavenging capacity (ORAC) assay. The complexes also showed promising results in the treatment of LS-174 colorectal adenocarcinoma. Cytoselectivity depended on the introduced modifications based on the introduction of acetylacetonate and trifluoroacetylacetonate ligands to CuAA and CuAT.

**Figure 28:**
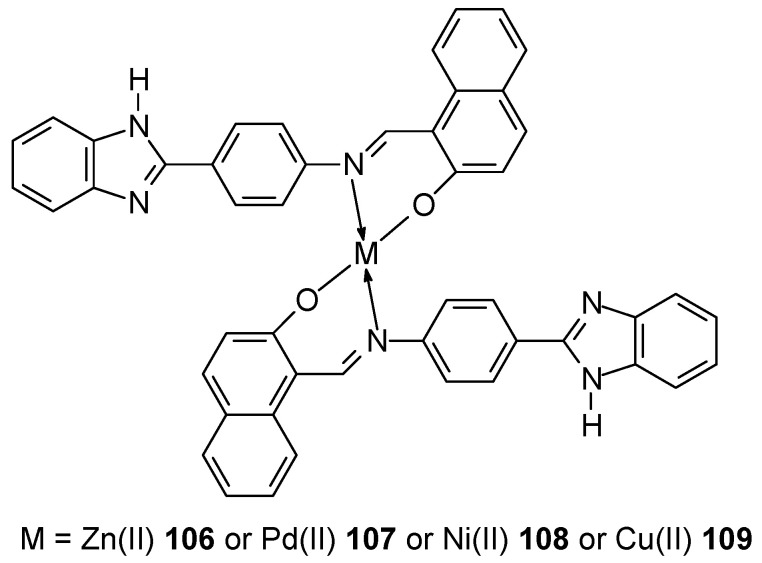
Structure of complexes obtained by Mahmood et al. [83].

Mahmood et al. [82] synthesized two new benzimidazole ligands: (*E*)-2-((4-(1H-benzo[d]imidazol-2-yl)phenylimino)methyl)-6-bromo-4-chlorophenol (L1) and (*E*)-1-((4-(1H-benzo[d]imidazol-2-yl)phenylimino)methyl)naphthalen-2-ol (L2) along with the corresponding them with Cu(II), Ni(II), Pd(II) and Zn(II) complexes for ligand L1 **102**–**105** and L2 **106**–**109** (Figure 28).

Studies conducted on breast cancer cell lines showed that cancer cells showed lower viability when treated with synthesized Schiff base complexes compared to standard drugs cisplatin and doxorubicin at the same concentration of the administered drug. The conducted analyses indicate that their mechanism of action is based on binding to DNA via intercalation and groove binding mode. This is evidenced by the binding constant value of 10^3^–10^5^ M^−1^, with the highest binding strength being for the nickel complex C_40_H_26_N_6_O_2_NiCl_2_Br_2_ (K_b_, 3.27 × 10^5^ M^−1^).

Feizpour et al. [83] resented the synthesis of new metal complexes (Mn(II), Fe(III), Ni(II), Cu(II), and Zn(II)) with a fluorescent ligand exhibiting antiproliferative activity (**110**–**114**). They have a structure with distorted octahedral geometry, where the center is surrounded by two ligands. They were obtained by the reaction of a Schiff base with the appropriate metal salts (Mn(HL)_2_Cl_2_, Fe(HL)_2_Cl_3_⋅3H_2_O, Ni(L)(HL)Cl⋅8H_2_O, Cu(HL)Cl_2_⋅4H_2_O, Zn(H_2_L)Cl_3_). The new ligand was obtained in a multi-step reaction by condensation in a 1:1 molar ratio of aldehyde (3-(3-formyl-4-hydroxybenzyl)-1-methyl-1H-imidazol-3-ium chloride) and 4-(1-naphthyl)-3-thiosemicarbazide. The syntheses of the substrates were previously described in the literature [84,85]. The most interesting results were obtained for the copper(II) ion complex. It showed activity against both human breast adenocarcinoma (MCF-7) and human liposarcoma (SW-872) cancer cells. The determined IC_50_ values were 127.6 ± 5.69 μM for MCF-7 and 35.66 ± 0.56 μM for SW-872, respectively. For comparison, comparative analyses were performed against paclitaxel (PTX) and cisplatin, for which the IC_50_ was 26.14 ± 1.66 μM (MCF-7), 2.58 ± 1.46 μM (SW-872), 59.78 ± 3.59 μM (MCF-7), and 10.06 ± 5.71 μM (SW-872), respectively. Promising results were also obtained for the nickel(II) ion complex, for which the IC_50_ against MCF-7 cells was 79.14 ± 1.01 μM. The main cause of cell death was apoptosis, and cell cycle studies showed that cell cycle arrest occurred in the G1 and S phases for the copper(II) ion complex, while for the zinc(II) ion complex in the G2 and G1 phases.

Mamta et al. [86] reported the microwave-assisted synthesis of zinc(II) ion complexes **115**–**117** with a macrocyclic Schiff base. These results were a continuation of earlier work [86,87,88,89]. Schiff bases were also synthesized classically by reacting 1,3-diphenyl-1,3-propanedione with 3,4-diaminotoluene for compound N_4_MacL_1_, 1,8-diaminonaphthalene for compound (N_4_MacL_2_), and 4-chloro-o-phenylenediamine for compound (N_4_MacL_3_). Both the classical method and microwave-assisted synthesis were also used to synthesize complexes with octahedral geometry. Better results were obtained using the latter technique. In addition to achieving better yields, the reaction time was also shortened from 10 to 12 h to about 12–15 min. They demonstrated the validity of using an innovative technology, which is enjoying increasing interest in the pharmaceutical community, accelerating the synthesis of new therapeutics [90,91]. The macrocyclic complex Zn(N_4_MacL_3_)Cl_2_ demonstrated the highest cytotoxic activity IC_50_ for the MCF-7 cell line, amounting to 7.40 ± 0.45 µM. Additionally, it showed activity against A549 (an epithelial cell line of human follicular adenocarcinoma), HT-29 (a cell line of human colon adenocarcinoma) of 2.23 ± 0.25 µM, 6.53 ± 0.28 µM, respectively. According to Tweedy’s chelation theory, the biological activity of the macrocyclic complexes was improved.

**Figure 29:**
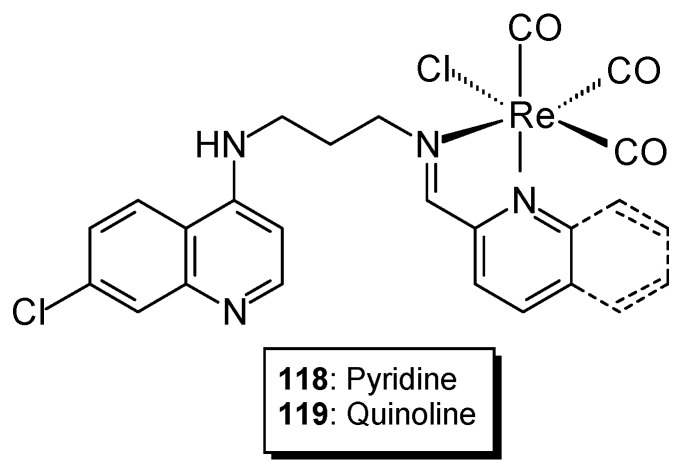
Structure of Re(I) complexes.

In the search for alternatives to platinum-based anticancer drugs, new synthesized compounds containing ruthenium, titanium, or gold have been utilized. Compounds containing transition metals such as iridium, osmium, and rhenium have also been synthesized. Studies have shown that Re(I) tricarbonyl complexes containing quinoline fragments exhibit cytotoxic activity against cancer cell lines. Studies of complexes prepared based on 4-aminoquinoline Schiff base ligands (containing iminoquinoline **118** or iminopyridine **119** with a [Re(CO)_3_Cl]^+^ core (Figure 29) indicate that metal complexation significantly affects the compound’s activity and demonstrates higher cytotoxic activity than cisplatin [92,93,94]. The IC_50_ of the iminoquinolyl complex was 6.82 ± 1.03 μM, while the iminopyridyl complex showed a value of 8.55 ± 1.08 μM. Western blot studies showed that the complexes lead to DNA damage and cell apoptosis.

Zinc(II) complexes with a quinoline system may be an alternative to rhenium(I) complexes **120**–**121**. Côrte-Real and co-workers [95] obtained complexes containing 8-hydroxyquinoline Schiff bases functionalized at the 2-position with 1-(3-aminopropyl)imidazole (HL_1_) or 1-(3-aminopropyl)-2-methyl-1H-imidazole (HL_2_). In contrast to other monomeric complexes, [Zn(L_1_)_2_]_n_ exhibited a one-dimensional 1D polymeric chain structure in which the bridging Schiff base ligands and Zn(II) cations were arranged alternately. The geometry of the complex indicates an octahedral structure. The cytotoxicity of the complexes was determined with particular emphasis on human breast cancer cells (MDA-MB-231, MDA-MB-453, MCF-7, BT549), as well as prostate (Du145), pancreas (Panc-1), lung (A549), and melanoma (A375) cell lines, compared to control cells (RPE-1 cells). The results obtained were worse than those for the positive control, which was cisplatin. The IC_50_ value for MCF-7 cells was 7.3 ± 2.4 µM for Zn(L_1_)_2_ and 6.7± 1.0 µM for Zn(L_2_)_2_, respectively, while for cisplatin it was 5.0 ± 0.4 µM. However, cisplatin causes significant damage to normal cells, while the tested Zn(II) complexes demonstrated effective eradication of cancer cells while sparing normal cells. These results are promising and prompt further research. Studies of the action of these complexes indicated that apoptosis was the primary cause of cell death. Simultaneously, these complexes were observed to effectively induce reactive oxygen species and cause double-stranded DNA breaks only in the presence of cytosolic molecules; this effect was not observed in cell-free systems.

**Figure 30:**
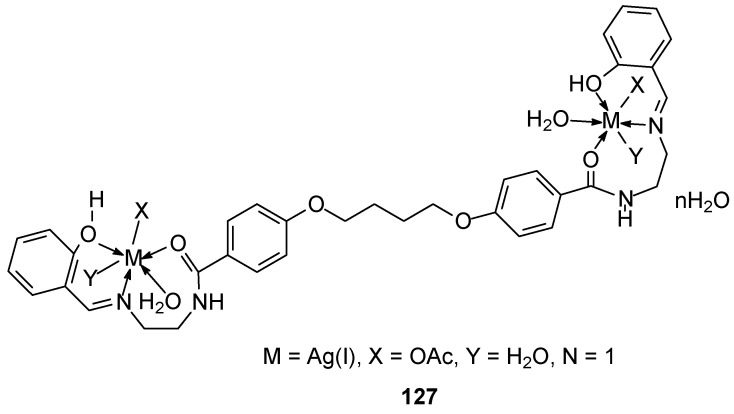
Ag(I) complexed with ether ligand synthesized by Faheem at al. [97].

Faheem and his colleagues [96] synthesized an ether ligand and its complexes **122**–**127** with metals such as Mn(II), Ni(II), Cu(II), Zn(II), Hg(II), and Ag(I) (Figure 30). Their biological studies revealed, among other properties, activity against the MCF-7 breast cancer cell line. The authors believe this activity stems from the ability of the selected complexes to bind to DNA.

**Figure 31:**
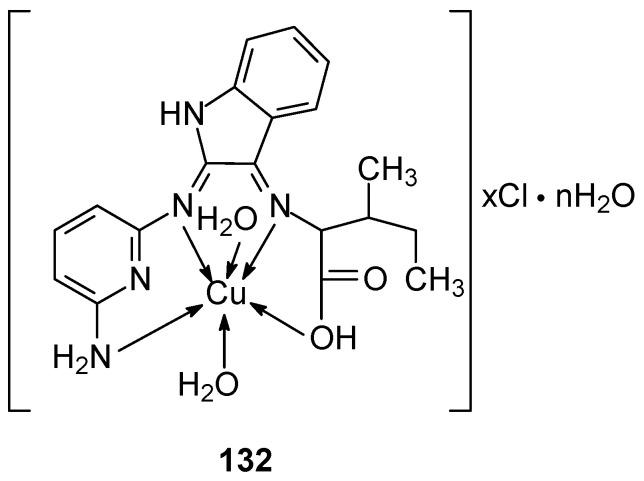
Structure of Cu(II) complex with Schiff base ligand (HL) [97].

The synthesis of new complexes **128**–**135** exhibiting inhibitory potential towards 1GS4, 2HQ6, 3DJD, and 5JPE receptors was described by Derafa et al. [97]. The ligand(cyclopenta-2,4-dien-1-yl)(cyclopenta-2,4-dien-1-yl)(1-((8-aminonaphthalen-1-yl)imino)ethyl) was synthesized from isatin, isoleucine, and 2,6-diaminopyridine. The complexes (Figure 31) were obtained by reaction with appropriate salts. The ligands were shown to form a non-negative four-dentate bond with the metal ion, most of which exhibited tetrahedral geometry, except for Ni(II), Co(II), and Zn(II) which exhibited octahedral geometry. The metal complexes exhibited activity against MCF-7 breast cancer cells compared to the free ligand. Additionally, increased antibacterial, antifungal and antiproliferative activity was observed [97]. The inhibition ratio for the complexes ranged from 74% to 86% at concentrations of 100 to 150 µg/mL against the human breast cancer cell line MCF-7. The determined IC_50_ values for the complexes ranged from 12 to 23.1 µg/mL, while for (cyclopenta-2,4-dien-1-yl)(cyclopenta-2,4-dien-1-yl) (1-((8-aminonaphthalen-1-yl)imino)ethyl the value was 23.1 µg/mL. The lowest value was determined for Co(II) equal to 12.0 µg/mL. Similar values were found for the Zn(II) and Mn(II) complexes, 14.0 µg/mL and 13.3 µg/mL, respectively, which indicates strong anticancer activity. The remaining complexes of Cu(II), Fe(III), Ni(II), Cr(III) and Cd(II) ions had IC_50_ values of 14.7, 15.9, and 17.9, respectively. 17.6, 19.1 and 20.3 µg/mL.

Another approach to anticancer drug design was presented by Ferreira et al. It was based on comparing the effect of the counterion on the anticancer activity of the obtained complexes **136**–**138**. Three Cu(II) complexes were obtained based on the ligand 1-(1H-benzimidazol-2-yliminomethyl)naphthalen-2-ol as the Schiff base (L). The differences in the complexes were based on the presence of the counterion ClO_4_^−^ in the complex ([Cu(L)(H_2_O)]ClO_4_), acetate in [Cu(L)(OAc)] and nitrate in [Cu(L)(NO_3_)] [98,99]. In vitro cytotoxicity studies showed moderate cytotoxicity of the [Cu(L)(OAc)] and [Cu(L)(NO_3_)] complexes towards MCF-7 cell lines. The [Cu(L)(NO_3_)] complex against MCF-7 showed lower IC_50_ values (IC_50_ = 56.5 ± 1.8 µM) compared to the free ligand (L) (IC_50_ = 97.3 ± 1.8 µM), [Cu(L)(H_2_O)]ClO_4_ (IC_50_ = 78.1 ± 1.7 µM) and [Cu(L)(OAc)] (IC_50_ = 63.9 ± 1.8 µM). The obtained results indicate that the type of counterion in the complex influences their activity against the MCF-7 cell line. The complex with nitrate as a co-ligand likely showed enhanced activity due to the presence of the nitrate group, which may facilitate interactions with biomolecules [100].

Ibrahim et al. [101] described the synthesis and characterization of new Schiff base ligands by reacting 3-nitrobenzaldehyde with thiourea in ethanol. The new ligand was used to complex copper(II) **139** and zinc(II) **140** ions. Biological studies showed that, in addition to antibacterial activity, ZnO and CuO nanoparticles exhibited activity against human MCF-7, SW620, and A549 cells, as confirmed by MTT assays. The use of the nanoparticles indicated that CuO nanoparticles exhibited the highest cytotoxic effect, surpassing ZnO nanoparticles. Additionally, CuO nanoparticles demonstrated greater antitumor efficacy than doxorubicin. This represents a new approach to the use of oxide nanoparticles and conjugated non-noble metal complexes of Schiff bases in the treatment of colon cancer. therapeutic. Table 3 summarizes the information contained in this chapter.

## 4. Conclusions

The high incidence and mortality rates associated with breast cancer in women prompt the search for new, effective, and selective drugs for this type of cancer. Metal complexes, particularly noble metals such as platinum, have played a significant role in anticancer therapy for years. Currently, complexes of metals such as Mn, Co, Fe, Ni, Cu, and Zn are increasingly being used. As published results demonstrate, complexes of these metals containing Schiff base ligands are gaining increasing interest due to their high therapeutic potential. Salicylic aldehyde derivatives are among the most frequently used ligands in recent years, due to the most common method for synthesizing Schiff bases, which involves reacting the aldehyde with a primary amine. They are characterized by good activity against the MCF-7 cell line and often exhibit better selectivity than the commonly used cisplatin. Notably, they also frequently exhibit cytotoxicity against other cancer cell lines. A second group of complexes with potential anticancer properties, which may become future drugs in breast cancer therapy, are metal complexes with sulfur-containing Schiff base ligands. Studies on the activity of these complexes synthesized over the past five years place them among the potential future anticancer drugs. The possibilities of using Schiff base complexes with various metals in the treatment of a number of different diseases have been studied for years, and as numerous research results from recent years show, they may in the near future constitute active substances of new drugs against breast cancer.

## Figures and Tables

**Table 1:** Summary of selected information on the activity of metal complexes with Schiff base ligands derived from salicylaldehyde.

Comp.	Ligand	Metal Ion	IC_50_ MCF-7	Other Tested Cell Lines	Other Tests	Ref.
**1**	4(N,N)-diethylaminosalicylaldehyde-4(N)-thiosemicarbazone [H_2_-DEAsal-tsc]	Ni(II)	5.37 ± 0.21 μM	A549 HeLa	ctDNA, BSA	[38]
**2**	4(N,N)-diethylaminosalicylaldehyde-4(N)-methylthiosemicarbazone [H_2_-DEAsal-mtsc]		4.91 ± 0.18 μM			
**3**	4(N,N)-diethylaminosalicylaldehyde-4(N)-ethylthiosemicarbazone [H_2_-DEAsal-etsc]		4.66 ± 0.22 μM			
**4**	4(N,N)diethylaminosalicylaldehyde-4(N)-phenylthiosemicarbazone [H_2_-DEAsal-ptsc]		5.69 ± 0.17 μM			
**5**	((*E*)-2-ethoxy-6((pyren-1-ylimino)methyl)phenol)	Zn(II)	12.742 ± 0.73 μg/mL			[39]
**6**		Cu(II)	5.661 ± 0.33 μg/mL			
**7**		Fe(III)	58.708 ± 3.37 μg/mL			
**8**		Cr(III)	16.895 ± 0.98 μg/mL			
**9**		Co(II)	21.141 ± 1.21 μg/mL			
**10**	salicylidene carbohydrazide	Cu(II)	2.22 ± 0.08 μM	MDA-MB-231	ctDNA, ROS, MTT	[40]
**11**	2-(1-methyl-1H-benzo[d]imidazol-2-yl)aniline with (3-formyl-4-hydroxybenzyl)triphenylphosphonium chloride	Cu(II)	25.00 ± 1.17 μM	A-549 HeLa	ctDNA, ROS, AO/EB, MTT	[41]
**12**	2-(1-methyl-1H-benzo[d]imidazol-2-yl)aniline with N,N-diethyl-N-(3-formyl-4-hydroxybenzyl)ethanaminium chloride		80.12 ± 0.016 μM	HaCaT	Molecular docking with DNA	
**13**	4-fluoro-N-((3-hydroxy-5-(hydroxymethyl)-2-methylpyridin-4-yl)methylene)benzohydrazide (PLFBH)	Cu(II)	15.30 ± 0.55 μM	HelLa A549	ctDNA	[42]
**14**	3,5-dichlorosalicylaldehyde and trans-1,2-diaminocyclohexane	Ni(II)	108.1 µg/mL		DPPH Docking with BSA Docking DNA, MTT test PAINS	[43]
**15**	N-salicylyl-L-tryptophan sodium salt	Fe(III)	4.3 ± 0.2 μM	MG-63 HT-29 L929	BSA, ctDNA, MTT test ROS	[44]
**16**			10.7 ± 2.5 μM			
**17**	2-Chloro-5-Nitrophenyl-(4,6-Dimethylpyrimidinyl)methanimine Schiff Base	Ru(III)	46.7 µM	T47D HCT116 HepG2	Annexin V/Propidium Iodide Staining for Apoptosis Assessment Expression Levels of Caspase 3, VEGF-A, mTOR, NF-kB, and SND1 by RT-PCR	[45]
**18**	potassium(E)-2-((5-bromo-2-hydroxybenzylidene)amino) 3-methylbutanoate	Cu(II)	17.13 ± 0.74 μM	A549 HeLa	MTT test DPPH	[46]
**19**			33.18 ± 1.14 μM			
**20**	4-bromo-2-[(E)-{[4-(2-hydroxyethyl)phenyl]imino}methyl]phenol	Fe(III)	60.00 µg/µL	HepG2	SAR ctDNA ROS	[47]
**21**		Cr(III)	37.00 µg/µL			
**22**	2-[(E)-{[4-(2-hydroxyethyl)phenyl]imino}methyl]-4-methoxy phenol	Cr(III)	37.00 µg/µL			
**23**		Mn(II)	3.00 µg/µL			
**4**	1,3-propanediamine with 2-hydroxy-4-methoxybenzaldehyde	Cu(II)	15.87 µM	HCT116 A549	Molecular docking	[48]
**25**			4.97 µM			
**26**			21.75 µM			
**27**	Condensation L-tryptophan with 5-chlorosalicyladehyde	Cu(II)		MDA-MB 231 MCF-10A	ctDNA, BSA, ROS, SRB test	[49]
**28**			4.31 ± 1.1 µM			
**29**	Condensation L-tryptophan with 3,5-chlorosalicyladehyde		2.38 ± 0.3 µM			
**30**	Ligand as Schiff bases—derivative of reaction L-tyrosine and salicylaldehyde	Co(II)	8.8 µg/mL		ctDNA DPPH radical scavenging activity	[50]
**31**		Ni(II)	2.8 µg/mL			
**32**		Zn(II)	2.5 µg/mL			
**33**		Cu(II)	4.2 µg/mL			
**34**	Condensation of 2,2-dimethyl-1,3-propanediamine with salicylaldehyde	Cu(II)	90 μM	HCT-116 A549	Molecular docking with DNA	[51]
**35**	Condensation of 2,2-dimethyl-1,3-propanediamine with 5-bromosalicylaldehyde		147.4 μM			
**36**	Condensation of 2,2-dimethyl-1,3-propanediamine with 3-methoxysalicylaldehyde		21.7 μM			
**37**	(E)-N′-(5-bromo-2-hydroxybenzylidene)isonicotinohydrazide	Co(II)	7.26 μg/mL		DPPH radical scavenging activity Antioxidant activity	[52]
**38**		Ni(II)	70.93 μg/mL			
**39**		Cu(II)	128.32 μg/mL			
**40**		Zn(II)	2.73 μg/mL			

**Table 2:** Summary of selected information on the activity of metal complexes with Schiff base ligands containing a sulfur atom.

Comp.	Ligand	Metal Ion	IC_50_ MCF-7	Other Tested Cell Lines	Other Tests	Ref.
**41**	[3,5-dimethyl-1-phenyl-4-(phenylselanyl)-1H-pyrazole]	Cu(II)	44 ± 11 (SI = 1.4) μM	V79 MRC-5 U2OS HepG2	DPPH	[53]
**42**	[3,5-dimethyl-1-phenyl-4-(phenylsulfur)-1H-pyrazole]		59 ± 2 μM			
**43**	4-((5-Bromo-2-hydroxybenzylidene)amino)-benzenesulfonamide	Ni(II)	-	OEC		[55]
**44**	4-((5-Bromo-2-hydroxybenzylidene)amino)-N-(1,3-thiazol-2-yl)benzenesulfonamide		11.2 ± 0.9 μM			
**45**	4-((3,5-Dibromo-2-hydroxybenzylidene)amino)-benzenesulfonamide		-			
**46**	4-((3,5-Dibromo-2-hydroxybenzylidene)amino)-N-(1,3-thiazol-2-yl)benzenesulfonamide		4.33 ± 0.5 μM			
**47**	4-((3,5-Diiodo-2-hydroxybenzylidene)amino)-N-(1,3-thiazol-2-yl)benzenesulfonamide		>100 μM			
**48**	2-((E)-(6-Ethoxybenzo[d]thiazol-2-ylimino)methyl)-4-chlorophenol	Zn(II)	37.67 μM	HeLa	ctDNA, Inhibition of RS formation, Antioxidant activity	[56]
**49**		Ni(II)	51.32 μM			
**50**		Co(II)	58.41 μM			
**51**		Cu(II)	67.59 μM			
**52**	SMDTC-glyoxal	Cu(II)	1.7 ± 0.1 µM	MDA-MB-231		[57]
**53**	SBDTC–glyoxal		>50 µM			
**54**	SMDTC–Butanedione		46 ± 1.0 µM			
**55**	SBDTC–Butanedione		11 ± 1.9 µM			
**56**	SMDTC–Pentadione		14 ± 2.1 µM			
**57**	SBDTC–Pentadione		>50 µM			
**58**	SMDTC–Hexadione		45 ± 2.3 µM			
**59**	SBDTC–Hexadione		7.3 ± 2.8 µM			
**60**	SMDTC–Heptadione		20 ± 1.5 µM			
**61**	SBDTC–Heptadione		>50 µM			
**62**	Sodium 2-hydroxy-3-methoxy-5-sulfonate-benzaldehyde-3-thiosemicarbazone	Cu(II)	4.1 ± 1.0 μM	HCT-15 LoVo HEK293 2008 MDA-MB-231 A431 PSN-1		[62]
**63**	Sodium 2-hydroxy-3-methoxy-5-sulfonate-benzaldehyde-4-ethyl-3-thiosemicarbazone		8.3 ± 0.2 μM			
**64**	Sodium 2-hydroxy-3-methoxy-5-sulfonate-benzaldehyde-3-methyl-thiosemicarbazone		8.9 ± 0.4 μM			
**65**	Sodium 2,3-dihydroxy -5-sulfonate-benzaldehyde-3-thiosemicarbazone		1.3 ± 0.4 μM			
**66**	Sodium 2,3-dihydroxy-3-methoxy-5-sulfonate-benzaldehyde-4-ethyl-3-thiosemicarbazone		1.7 ± 1.0 μM			
**67**	Sodium 2-hydroxy-3-methoxy-5-sulfonate-benzaldehyde-3-thiosemicarbazone	Ni(II)	>100 μM			
**68**	Sodium 2-hydroxy-3-methoxy-5-sulfonate-benzaldehyde-3-thiosemicarbazone	Zn(II)	>100 μM			
**69**	(E)-2-morpholino-N-(thiophen-2-ylmethylene)ethanamine	Co(II)	4.0 ± 1.06 μM			[68]
**70**		Cu(II)	5.9 ± 0.23 μM			
**71**		Zn(II)	3.3 ± 0.01 μM			
**72**		Cd(II)	4.0 ± 1.06 μM			
**73**	curcumin and synthesized 2-amino-3-carboxyethyl-4,5-dimethylthiophene	Mn(II)	<10 μg/mL	K-562	DPPH, Antioxidant activity	[69]
**74**		Co(II)	<10 μg/mL			
**75**		Ni(II)	<10 μg/mL			
**76**		Cu(II)	>80 μg/mL			
**77**		Zn(II)	<10 μg/mL			
**78**	Hatc-Ch: 2-Acetylpyridine-4-cyclohexyl-3-thiosemicarbazone	Zn(II)	9.43 µM	MDA-MB-453 MDA-MB-231 MCF10A HUVEC HFF MCF10A HUVEC HFF		[71]
**79**	Hatc-Et: 2-Acetylpyridine-4-ethyl-3-thiosemicarbazone		18.49 µM			
**80**	Hsc: 2-Acetylpyridine-semicarbazone		19.34 µM			
**81**	Hhz: 2-Acetylpyridine-furanoylhidrazone		10.41 µM			
**82**	(Z)-2-((E)-1-(2-(4-chlorophenyl)hydrazinylidene)propan-2-ylidene)-N-phenylhydrazine-1-carbothioamide	Fe(III)	20 µg/mL			[72]
**83**		Co(II)	23 µg/mL			
**84**		Cu(II)	10.5 µg/mL			
**85**	(E)-7-methoxy-N-(4-methoxybenzylidene)benzo[d]-thiazol-2-amine	Cu(II)	12 ± 0.03 (μg ± SD)	Hela Hep2 HepG2	Antioxidant activity by DPPH method, DFT calculation, Molecular docking	[76]
**86**		Zn(II)	24 ± 0.15 (μg ± SD)			
**87**		Ni(II)	37 ± 0.05 (μg ± SD)			
**88**		Co(II)	43 ± 0.06 (μg ± SD)			
**89**	1,5-bis(2-methoxyanisaldehyde)thiocarbohydrazine	Sn(II)	263.50 ± 38.89 µM	A549 HeLa U87 T47D MDA-MB-231 MDA-MB-453 BT-549 PANC1 HT-29 HCT116 SW480 SW620 CACO2 RAW	SRB, DPPH	[77]
**90**		Zn(II)	47.69 ± 3.32 µM			
**91**		Fe(II)	183.20 ± 6.72 µM			
**92**	1,5-bis (4-methoxyanisaldehyde)thiocarbohydrazine	Sn(II)	434.64 ± 35.44 µM			
**93**		Zn(II)	157.17 ± 7.74 µM			
**94**		Fe(II)	135.06 ± 6.84 µM			

**Table 3:** Summary of selected information on the activity of metal complexes with Schiff base ligands that are neither derived from salicylaldehyde nor contain sulfur.

Comp.	Ligand	Metal Ion	IC_50_ MCF-7	Other Tested Cell Lines	Other Tests	Ref.
**95**	Cyclohexane-1,2-diamine, 2,6-diformyl-4-methylphenol	Co(II)	16.81 ± 1.33 μM	LS-174 MCR-5		[80]
**96**	[Cu(acac2 en)]	Cu(II)	17.53 ± 2.83 μM		ROS, Molecular docking interaction with HSA	[81]
**97**	[Cu(phacac2 en)]					
**98**	[Cu(tfacac2 en)]		21.29 ± 2.55 μM			
**99**	[Cu(acac tfacac en)]					
**100**	[Cu(acac tfacac en)]		30.02 ± 2.05 μM			
**101**	[Cu(acac phacac en)]					
**102**	(E)-2-((4-(1H-benzo[d]imidazol-2-yl)phenylimino)methyl)-6-bromo-4-chlorophenol (L1)	Cu(II)			Molecular docking	[82]
**103**		Ni(II)	1.89 LD50 mg/mL			
**104**		Pd(II)				
**105**		Zn(II)				
**106**	(E)-1-((4-(1H-benzo[d]imidazol-2-yl)phenylimino)methyl)naphthalen-2-ol (L2)	Cu(II)	0.129 LD50 mg/mL			
**107**		Ni(II)				
**108**		Pd(II)	3.09 LD50 mg/mL			
**109**		Zn(II)				
**110**	Condensation of aldehyde (3-(3-formyl-4-hydroxybenzyl)-1-methyl-1H-imidazol-3-ium chloride) and 4-(1-naphthyl)-3-thiosemicarbazide	Mn(II)	257.1 ± 2.90 μM	SW-872		[83]
**111**		Fe(III)	193.4 ± 2.57 μM			
**112**		Ni(II)	79.14 ± 1.01 μM			
**113**		Cu(II)	127.6 ± 5.69 μM			
**114**		Zn(II)	206.9 ± 5.61 μM			
**115**	N4MacL1	Zn(II)	10.23 ± 0.41 µM 9.78 ± 0.32 µM 7.40 ± 0.45 µM	A549 HT-29		[86]
**116**	N4MacL2					
**117**	N4MacL3					
**118**	N1-(7-chloroquinolin-4-yl)propane-1,3-diamine	Re(I)	8.55 ± 1.08 μM	MDA-MB-231 FG-0	Molecular docking	[92]
**119**	7-Chloro-N-(3-((quinolin-2-ylmethylene)amino)propyl)quinolin-4-amine		6.82 ± 1.03 μM			
**120**	functionalized at the 2-position with 1-(3-aminopropyl)imidazole (HL1)	Zn(II)	7.3 ± 2.4 µM			[95]
**121**	functionalized at the 1-(3-aminopropyl)-2-methyl-1H-imidazole (HL2).		6.7 ± 1.0 µM			
**122**	combination of two moles of salysaldehyde 4,4′-(butane-1,4-diylbis(oxy))bis(N-(2-aminoethyl) benzamide)	Mn(II)	data on the chart	HepG-2	Molecular docking	[96]
**123**		Ni(II)				
**124**		Cu(II)				
**125**		Zn(II)				
**126**		Hg(II)				
**127**		Ag(I)				
**128**	(cyclopenta-2,4-dien-1-yl)(cyclopenta-2,4-dien-1-yl) (1-((8-aminonaphthalen-1-yl)imino)ethyl)	Cr(II)	19.1 mg/mL		Molecular docking	[97]
**129**		Fe(III)	13.3 mg/mL			
**130**		Mn(II)	15.9 mg/mL			
**131**		Cu(II)	12.0 mg/mL			
**132**		Cd(II)	17.6 mg/mL			
**133**		Co(II)	14.7 mg/mL			
**134**		Zn(II)	14.0 mg/mL			
**135**		Ni(II)	20.3 mg/mL			
**136**	1-(1H-benzimidazol-2-yliminomethyl)naphthalen-2-ol	([Cu(L)(H_2_O)]ClO_4_)	78.1 ± 1.7 µM			[100]
**137**		[Cu(L)(OAc)]	63.9 ± 1.8 µM			
**138**		[Cu(L)(NO_3_)]	56.5 ± 1.8 µM			
**139**	new Schiff base ligands by reacting 3-nitrobenzaldehyde with thiourea	Cu(II)	data on the chart	SW620 A549	Antioxidant activity	[101]
**140**		Zn(II)				

## Data Availability

The original contributions presented in this study are included in the article. Further inquiries can be directed to the corresponding authors.

## Abbreviations

bpy	2,2′-Bipyridine
BSA	Bovine Serum Albumin
Cl8HQ	5-chloro-8-quinolinol
ctDNA	circulating tumor DNA
dppm	1,1′-bis(diphenylphosphino)methane
DEAsal	4(N,N)-diethylaminosalicylaldehyde
DFT	Density Functional Theory
DPPH	2,2-diphenylo-1-picrylhydrazyl
etsc	ethylthiosemicarbazone
FDA	Food and Drug Administration
8HQ	8-hydroxyquinoline
IC_50_	IC_50_ is defined as the concentration of a drug required for 50% inhibition of a biological or biochemical function
LDH	Lactate dehydrogenase
mtsc	methylthiosemicarbazone
MTT	colorimetric assay for assessing cell metabolic activity
phen	1,10-phenanthroline
ptsc	phenylthiosemicarbazone
py	pyridine
ROS	Reactive Oxygen Species
sal	salicylaldehyde
SI	Selectivity index
SRB	sulfate-reducing bacteria
tsc	thiosemicarbazone
WHO	World Health Organization
